# Gaming addiction, problematic gaming and engaged gaming – Prevalence and associated characteristics

**DOI:** 10.1016/j.abrep.2020.100324

**Published:** 2020-12-05

**Authors:** Frida André, Niroshani Broman, Anders Håkansson, Emma Claesdotter-Knutsson

**Affiliations:** a Medical Faculty, Department of Clinical Sciences Lund, BMC F12, Sölvegatan 19, 221 84 Lund, Sweden

**Keywords:** Gaming, Gaming addiction, GAS, Risk factors, ICD 11, International Classification of Diseases, DSM-5, Diagnostic and Statistical Manual of Mental Disorders (DSM–5), ESA, Entertainment Software Association, RSP, Remaining Study Participants

## Abstract

**Introduction:**

Gaming disorder was included in the 11th revision of the International Classification of Diseases (ICD 11) and the Diagnostic and Statistical Manual of Mental Disorders (DSM-5) included Internet Gaming Disorder as a tentative diagnosis. Most scholars agree upon the potential risk for pathological use of video games. The primary aim of this study was to investigate the prevalence of engaged gamers, problem gamers and addicted gamers. The secondary aim was to describe these groups in terms of gender, age, social satisfaction, psychological wellbeing and hours spent chatting on internet/social media.

**Methods:**

We used survey-based data for this population-based research. The data was collected online in two different settings in 2017. In total 2075 participants were included.

**Results:**

4.5 percent met the criteria for highly engaged gaming, 5.3 percent were shown to be problem gamers and 1.2 percent met the cut off for game addiction. Young age, hours chatting on internet/social media, experiencing loneliness and considering seeking treatment for psychological distress were associated with both engaged, problematic and addictive gaming. Male gender was associated to problematic and addictive gaming. Hours spent chatting showed a greater correlation to problem/addictive gaming than to engaged gaming.

**Conclusion:**

The results of this study indicate that both highly engaged gamers, problem gamers and addicted gamers all experience loneliness and psychological distress to a greater extent than the remaining study participants. This adds to the knowledge of prevalence and features of gaming disorder. Additionally, preferably longitudinal research is needed in order to understand causality.

## Highlights

• This study showed a prevalence measure of addicted gamers as 1.2 percent.
• Male gender was associated to problematic and addictive gaming.
• Young age was associated with both engaged-, problem- and addictive gaming.
• Hours spent on chatting was associated with both engaged-, problem- and addictive gaming.
• Loneliness was associated with both engaged-, problem- and addictive gaming.

## 1 Introduction

During recent decades video gaming has become one of the most popular recreational activities. The video game industry has grown to a turnover of nearly a $100 billion worldwide (Griffiths et al., 2015, Kiraly et al., 2018). The Entertainment Software Association (ESA) showed that 75 percent of all US households have at least one person who plays video games and 70 percent of those under 18 regularly play video games (Entertainment Software Association, 2018). Though the benefits of gaming have been reported; with improved cognitive processes such as task-switching, attentional control and processing speed (Nuyens, Kuss, Lopez-Fernandez, & Griffiths, 2018), most scholars agree on the potential risk for pathological use of video games. A minority of the great number of individuals engaging in gaming seemingly do so to an extent or manner that causes them negative consequences (Ferguson, Coulson, & Barnett, 2011).

Gaming disorder was included in the 11th revision of the International Classification of Diseases (ICD 11), defined as a gaming behaviour of sufficient severity to result in significant impairment in areas of function. Gaming disorder is further characterized by impaired control and continuance despite the occurrence of undesirable effects (WHO, 2018). The Diagnostic and Statistical Manual of Mental Disorders (DSM-5) identifies Internet Gaming Disorder as a condition necessitating further clinical experience and research before inclusion as a formal disorder (American Psychiatric Association, 2013). Some scholars consider the application of gaming disorder as a clinical diagnosis to be premature and raise concerns about the risk of pathologizing normal behaviour (Aarseth, Bean, & Boonen, 2017). Others express an obligation to systematically distinguish between engagement and addiction in order to avoid such implications (Brunborg et al., 2013, Brunborg et al., 2015, Charlton and Danforth, 2007).

**Fig. 1:**
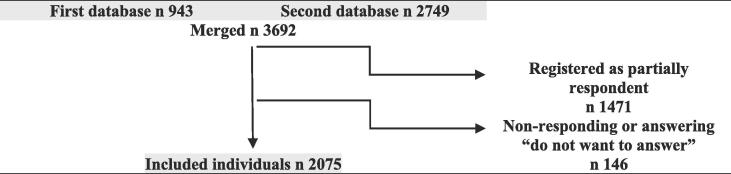
Inclusion procedure.

The GAS (Game Addiction Scale) is one of the most frequently used questionnaires for game addiction (Brunborg et al., 2013, Brunborg et al., 2015, Costa et al., 2019, Khazaal et al., 2016, King et al., 2020, Lemmens et al., 2009, Lin et al., 2019, Mentzoni et al., 2011, Wittek et al., 2015). The scale was theoretically based on the DSM-5 criteria for pathological gambling, namely; salience, tolerance, mood modification, withdrawal, relapse, conflict and problems. (Lemmens et al., 2009) The DSM-5 requires half (or more) of their criteria to be met when diagnosing pathological gamblers. Scholars within the field of gaming on the other side suggests a ranking of the criteria in order to separate highly engaged, and possibly less destructive gaming from problematic or addictive gaming. They describe how the criteria tolerance, mood modification and cognitive salience rather associate to high engagement and not necessarily to addiction, while the opposite applies for the criteria withdrawal, relapse, conflict and problems (Brunborg et al., 2013, Charlton and Danforth, 2007, Ferguson et al., 2011, Lehenbauer-Baum and Fohringer, 2015, Lehenbauer-Baum et al., 2015, Snodgrass et al., 2019). Hence research suggests application of the “core approach”, a system that distinguishes highly engaged gamers from problem- and addicted gamers by accentuating the latter criteria (withdrawal, relapse, conflict and problems) in order to estimate a precise and relevant prevalence, whereas a potential diagnosis of game addiction is expected to relate to interference and comorbidity rather than engagement (Brunborg et al., 2013, Brunborg et al., 2015, Charlton and Danforth, 2007) (see Fig. 1).

A great diversity in prevalence has been reported due to measurement approach and diagnostic criteria, in turn affecting the association with negative outcome such as mental health or social difficulties (Brunborg et al., 2013, Brunborg et al., 2015, Ferguson et al., 2011). Stevens et al. report in a meta-analysis that the prevalence of pathological gaming worldwide stands at 3.05 percent (Ferguson et al., 2011). Despite nearly two decades of research, there is still debate and controversy in the field regarding diagnostic criteria. The lack of consensus affects the reports of prevalence but also the comorbidity estimates and hence the mediated relevance of the condition (Aarseth et al., 2017, Ferguson et al., 2011) (see Table 1, Table 2, Table 3, Table 4, Table 5, Table 6).

**Table 1:** Excluded individuals.

Basic demographics		
	Total no.	Percent
Gender		
Male	98	47.1
Female	107	51.4
Transgender	3	1.4
Total*	208	12.9
Age, Years		
15–18	48	14.1
19–24	60	17.6
25–29	40	11.8
30–39	85	25.0
40–49	52	15.3
50–59	30	8.8
60-	25	7.4
Total**	340	21.0

* Numbers and percent of excluded individuals responding on gender items.

** Numbers and percent of excluded individuals responding on age items.

**Table 2:** Characteristics.

	**First database**		**Second database**		**Current study**	
	Total no.	Percent	Total no.	Percent	Total no.	Percent
Gender						
Male	260	52.5	770	50.7	1030	49.6
Female	292	46.8	747	49.2	1039	50.1
Transgender	4	0.7	2	0.1	6	0.3
Age, Years						
15–18	43	7.7	60	3.9	103	5.0
19–24	123	22.1	170	11.2	293	14.1
25–29	64	11.5	152	10.0	216	10.4
30–39	127	22.8	268	17.6	395	19.0
40–49	90	16.2	276	18.2	366	17.6
50–59	61	11.0	291	19.2	352	17.0
60-	48	8.6	302	19.9	350	16.9
Gaming behavior						
Addicted	5	0.9	20	1.3	25	1.2
Problem	57	10.3	52	3.4	109	5.3
Engaged	34	6.1	60	3.9	94	4.5
RSP-group	460	82.7	1387	91.3	1847	89

**Table 3:** Prevalence of gaming categories within subgroups.

	**Highly engaged gamers**		**Problem gamers**		**Addicted gamers**		**RSP-group**
	% (n)	p-value*	% (n)	p-value*	% (n)	p-value*	% (n)
Overall frequency	4.5 (94)		5.3 (109)		1.2 (25)		89.0 (1847)
Male gender							
Yes	3.9 (40)	0.211	6.4 (66)	0.021	1.6 (16)	0.140	88.2 (908)
No	5.2 (54)		4.1 (43)		0.9 (9)		89.9 (939)
Ever considered seeking treatment							
Yes	8.0 (59)	<0.001	7.6 (56)	<0.001	1.6 (12)	0.110	82.7 (607)
No	2.6 (35)		4.0 (53)		1.0 (13)		92.5 (1240)
Enough friends							
Yes	3.8 (63)	0.001	4.5 (75)	0.001	1.1 (19)	0.437	90.6 (1551)
No	7.7 (31)		8.4 (34)		1.5 (6)		82.4 (332)
Age							
15–18**	5.8 (6)	0.026	14.6 (15)	<0.001	2.9 (3)	0.053	76.7 (79)
19–24**	8.2 (24)	<0.001	10.6 (31)	<0.001	1.4 (4)	0.505	79.9 (234)
25–29**	11.6 (25)	<0.001	6.9 (15)	0.003	0.9 (2)	0.975	80.5 (174)
30-***	2.7 (39)	<0.001	3.2 (48)	<0.001	1.1 (16)	0.278	93.0 (1360)
Hours chatting on internet/social media							
<1–4	4.1 (81)	<0.001	4.5 (88)	<0.001	0.6 (12)	<0.001	90.7 (1775)
>4	10.9 (13)		17.6 (21)		10.9 (13)		60.5 (72)

* Compared with RSP-group.

** Compared with frequencies within the age group 30 years of age or older.

*** Compared with frequencies within all of the younger age categories.

**Table 4:** Problem/addicted gamers vs highly engaged gamers and the RSP-group.

**Problem/addicted gamers vs the remaining**		
	% (n)	*p-value*
Overall frequency	6.5 (134)	
Male gender		
Yes	8.0 (82)	0.006
No	5.0 (52)	
Ever considered seeking treatment		
Yes	9.3 (68)	<0.001
No	4.9 (66)	
Enough friends		
Yes	5.6 (94)	0.002
No	9.9 (40)	
Age		
15–18*	17.5 (18)	<0.001
19–24*	11.9 (35)	<0.001
25–29*	7.9 (17)	0.025
30-**	4.4 (64)	<0.001
Hours chatting on internet/social media		
<1–4	5.1 (100)	<0.001
>4	28.6 (34)	

* Compared with frequencies within the age group 30 years of age or older.

** Compared with frequencies within all of the younger age categories.

**Table 5:** Problem/addicted gamers vs both highly engaged gamers and the RSP-group.

**Problem/addicted gamers vs highly engaged gamers and RSP-group**	**OR**	**95% CI**	***p-*value**
Male gender			
Yes	2.737	1.826–4.104	<0.001
No	1		
Age			
15–18	1		
Per increase in age range	0.653	0.544–0.782	<0.001
Enough friends			
Yes	0.652	0.429–0.991	0.045
No	1		
Ever considered seeking treatment			
Yes	1.939	1.310–2.871	0.001
No	1		
Hours chatting on internet/social media			
>4	5.299	3.237–8.673	<0.001
<4	1

**Table 6:** Problem/addicted gamers vs engaged gamers.

**Problem/addicted gamers vs engaged gamers**	**OR**	**95% CI**	***p-*value**
Male gender			
Yes	2.066	1.170–3.647	0.012
No	1		
Age			
15–18	1		
Per increase in age range	1.001	0.760–1.319	0.992
Enough friends			
Yes	1.010	0.544–1.876	0.975
No	1		
Ever considered seeking treatment			
Yes	0.751	0.413–1.365	0.347
No	1		
Hours chatting on internet/social media			
>4	2.262	1.058–4.833	0.035
<1–4	1		

The addictive gaming has been associated to anxiety, depression, social phobia/anxiety, low social competence, low self-esteem, sleeping disorders and loneliness. It has also been associated to other computer based behavioural addictions such as Internet addiction and Facebook addiction (Andreassen et al., 2013, Gonzalez-Bueso et al., 2018, Lam, 2014, Lemmens et al., 2011, Pontes, 2017). Most of the research reports a higher prevalence of pathological gaming in males (Gonzalez-Bueso et al., 2018) and among teenagers and adolescents (Mentzoni et al., 2011, Wittek et al., 2015). A considerable part of previous research focuses on subpopulations such as young individuals or samples of a specific group of gamers. (Charlton and Danforth, 2007, Ferguson et al., 2011, Griffiths et al., 2015).

The primary aim of this study was to investigate the prevalence of engaged gamers, problem gamers and addicted gamers, using the core approach. The secondary aim was to describe how these groups of gamers differentiate in terms of gender, age, social satisfaction, psychological wellbeing and hours chatting on internet/social media.

## 2 Material and methods

### 2.1 Study procedure

The present study was designed as a web-based questionnaire. The data was collected online in two different settings in 2017. Both settings addressed problematic gaming, Internet use and gambling in the general population with the aim, among others, to study these behavioural addictions in relation to sexual minority status (Broman and Hakansson, 2018, Karlsson et al., 2019). The dataset was distributed online and presented as a self-test for problem gaming and gambling, targeting individuals above 15 years of age. Two Swedish universities assisted in distributing the survey online among students and staff and the survey was promoted through online advertising in social media as well as in Swedish news media.

The data collection was designed in collaborated with a marketing survey company in order to set up the questionnaire online, to handle the incoming data and to ensure anonymity blocking the IP addresses when collecting the data (Broman & Hakansson, 2018). During the first data collection the questionnaire was presented in Swedish yet with the option to choose from a range of minority languages. In the second data collection the questionnaire was presented in Swedish only. The first material was merged with the second database. This was possible as both of them used the measurement needed for the aimed prevalence estimate and addressed the same potential correlates.

The measures were based on self-reporting including demographics such as gender and age. Structured and well-established measuring tools were used for the problem behaviours of interest (problematic gambling, gaming and internet use) and the answering of these were mandatory while other questions were optional. The survey addressed social isolation – whether the individual felt that they had a sufficient number of friends to spend time with or whether they experienced feelings of loneliness and a desire to have more friends. The respondents were asked whether they had felt the need to seek treatment because of psychological health problems (*Yes, No or Do not want to answer*) and how many hours per day they spent on communication through social media, online chatting, Skype, WhatsApp or similar services (*<1, 1*–*2, 2*–*3, 4*–*3 or more than 4*).

### 2.2 Measures

#### 2.2.1 Game addiction scale

For the assessment of gaming behaviour, the seven-item version of the Gaming Addiction Scale (GAS) was used (Lemmens et al., 2009). The scale was constructed by Lemmens et al. in order to reflect components of addiction as well as the consequences thereof, namely: salience, tolerance, mood modification, relapse, withdrawal, conflict and problems (Griffiths, 2005, Lemmens et al., 2009). Each question covers one criterion, answered on a five-point continuum scale: 1 (never), 2 (rarely), 3 (sometimes), 4 (often), 5 (very often) and should according to the developer be accounted as endorsed when rated 3 or higher (Lemmens et al., 2009).

Aiming to distinguish level of severity within the group of gamers, the core approach was applied whereby the individuals meeting all of the core criteria (relapse, withdrawal, conflicts and problems) constituted the group addicted gamers. The respondents that endorsed 2–3 of the core criteria but none of the peripheral criteria (salience, tolerance, mood-modification) were grouped as problem gamers and those that endorsed all 3 of the peripheral criteria but not more than 1 of the core-criteria were grouped as engaged gamers (Brunborg et al., 2013, Brunborg et al., 2015, Wittek et al., 2015). Those who remained comprised the fourth and contrasting group, hereafter named remaining study participants (RSP). The RSP-group included individuals without gaming behaviour and individuals with gaming behaviour below the cut off for highly engaged gaming. The chosen methodology assumes that several core criteria being met implies a problematic use of video game which does not rule out a simultaneous endorsement of peripheral criteria. However, the group of engaged gamers is here considered as less problematic as they did not endorse more than one of the core criteria.

Since both the problem gamers and the addicted gamers were assumed to be associated with more severe gaming behaviour as well as more negative outcomes (Brunborg et al., 2013, Brunborg et al., 2015, Charlton and Danforth, 2007), these two groups also constituted one combined group (2–4 endorsed core criteria) enabling analyses against the rest of the respondents (=<3 endorsed core criteria).

#### 2.2.2 Gender

The respondents could report male, female or transgender as their gender identity. As male gender was supposed to be associated with pathological gaming (Gonzalez-Bueso et al., 2018, Mentzoni et al., 2011), a binary variable was created in which male gender was coded as 1 and female and transgender (non-male gender) were coded as 0.

#### 2.2.3 Age

Respondents age was a matter of interest since young age was thought to be associated with pathological gaming (Mentzoni et al., 2011, Wittek et al., 2015). Out of the originally seven age categories (15–18, 19–24, 25–29, 30–39, 40–49, 50–59 and 60 years of age or older), four binary variables were created (15–18, 19–24, 25–29, 30–) out of which the respondents reporting 15–18 years of age were coded as 1 and those reporting 30 years or older (30–39, 40–49, 50–59 and 60 years of age or older) were coded as 0, etcetera. The fourth variable composed of those reporting 30 years of age or older, coded as 1 and all of those reporting a younger age coded as 0.

#### 2.2.4 Ever considered seeking care

The respondents were asked whether they ever had felt the need to seek treatment for to psychological health complaints and could answer either *Yes, No or Do not want to answer*. As low psychological well-being has been associated with pathological gaming (Khazaal et al., 2016, Lemmens et al., 2011, Przybylski et al., 2017), a binary variable was created in which *Yes* was coded as 1 and the *No* as well as *Do not want to answer* as 0.

#### 2.2.5 Enough friends

Previous research has found an association between low social competence and loneliness with pathological gaming (Khazaal et al., 2016, Lemmens et al., 2011). In order to address social isolation, the respondents were asked whether they felt that they had a sufficient number of friends to spend time with, too many, or whether they experienced feelings of loneliness and a desire to have more friends. Those reporting feelings of loneliness were coded as 1 and the remaining respondents were coded as 0.

#### 2.2.6 Hours chatting on internet/social media

It has been hypothesized that excessive use of social media could be positively associated with pathological gaming (Andreassen et al., 2013, Pontes, 2017), such that the measure of time spent on social media was included in this investigation. There is no consensus regarding how many hours spent on social media that is pathological but several researchers suggest a cut-off around three-four hours(Jagtiani et al., 2019, Kaur et al., 2018, Strong et al., 2018). The respondents were asked about how many hours per day they usually spent on communication with others through social media - including chat functions within online games, WhatsApp, Skype or corresponding services, and were able to answer *Less than one hour, 1*–*2 h, 2*–*3 h, 3*–*4 h* or *More than 4 h*. A binary variable was created in which *More than 4 h* was coded as 1 whereas reporting of anything less was coded as 0.

### 2.3 Statistics

Estimates of frequencies and percentages as well as statistical analysis were performed in SPSS (IBM SPSS statistics version 24). The Chi-square test was used for statistical association between frequencies of engaged gamers, problem gamers and addicted gamers within subgroups such as gender, age categories, individuals experiencing feelings of loneliness, individuals experiencing the need to seek treatment for psychological distress and groupings based on hours chatting on internet/social media, in comparison to the RSP-group. The groups based on gender, age categories, the experience of loneliness, psychological distress and hours spent chatting were further analysed in a logistic regression using problem/addictive gaming as the outcome and compared separately with both engaged gamers and the RSP-group.

## 3 Results

### 3.1 Sample characteristics

After merging the two files the sample consisted of 3692 individuals. Only those registered as complete respondents (2777) were included. Among the complete respondents there were still a number of individuals who had abstained from answering some of the questions of interest or answered them with *Do not want to answer*– those were also excluded. The exclusion resulted in 2075 remaining individuals, of which 49.6 percent were male, 50.1 percent female and 0.3 percent transgender. A majority were aged 30 years or older and the smallest number (103 individuals, 5 percent) were seen in the age category 15–18. The excluded individuals were seemingly evenly distributed in terms of gender and showed a similar age distribution as the included population though the excluded group contained a greater proportion of the youngest individuals and a smaller proportion the oldest individuals.

### 3.2 Prevalence

The respondents who endorsed all four of the core criteria and consequently met the addiction cut-off comprised 1.2 percent. The problem gamers were 5.3 percent, the engaged gamers 4.5 percent and the respondents who met the cut off for at least problem gaming and at most addictive gaming, composed 6.5 percent.

### 3.3 Highly engaged gamers

Being an engaged gamer, was significantly associated with reporting a need to seek treatment for psychological health complaints in comparison to the RSP-group who denied such consideration. Engaged gaming was also associated to the reporting of feelings of loneliness. The engaged gamers were significantly overrepresented in the age ranges 15–18, 19–24 and 25–29, in comparison to the respondents of age 30 years or older. The engaged gamers were also shown to be significantly overrepresented in the subgroup that spent more than four hours per day on communication with others through social media/internet.

### 3.4 Problem gamers

When comparing frequencies of problem gamers with the RSP-group within subgroups, the former was revealed to be significantly overrepresented amongst respondents of male gender, amongst individuals who had considered seeking treatment, amongst individuals assenting feelings of loneliness, and in all of the younger age ranges, in comparison to the respondents 30 years of age or older. The problem gamers were also shown to be overrepresented amongst the respondents who spent more than four hours chatting on internet/social media.

### 3.5 Addicted gamers

The addicted gamers were shown to be significantly overrepresented amongst the respondents assenting that they spent more than four hours per day on communication with others through social media/internet, when compared with the RSP-group.

### 3.6 Problem/addicted gamers versus highly engaged gamers and the RSP-group

The respondents endorsing 3–4 of the core criteria and hence meeting the cut off for problem gaming or addiction, were shown to be significantly overrepresented amongst all of the subgroups of interest when compared with the other respondents (highly engaged gamers and the RSP-group). Thus, they were more common amongst the male respondents, those who considered seeking treatment for psychological health complaints, those who experienced feelings of loneliness, and amongst the younger age ranges, as well as amongst the individuals spending more than four hours chatting on internet/social media. The regression analysis presented corresponding associations in which spending more than four hours on chatting showed the most powerful correlation of problem-/ addictive gaming, elevating the probability 5.3 times.

### 3.7 Problem/addicted gamers versus engaged gamers

When comparing the probability of problem/addicted gaming with engaged gaming in a regression analysis, male gender and spending more than four hours chatting on internet/social media increased the probability of more adverse gaming significantly.

## 4 Discussion

The present study contributes to the knowledge about pathological gaming through an empirical as well as theoretically based prevalence measure of game addiction (Brunborg et al., 2013, Brunborg et al., 2015, Lemmens et al., 2009). Previous research in this field is scarce and rather inconsequential in terms of measurement approach and attitudes towards the tentative diagnosis (Ferguson et al., 2011). The DSM-5 requests more research before inclusion of the disorder and as the gaming industry grows and currently engages a wide range of the population, research needs to keep up with development (American Psychiatric Association, 2013, Entertainment Software Association, 2007, Entertainment Software Association, 2018). An increasing quantity of scholars emphasize a distinction between engagement and addiction regarding gaming research and a ranking of the criteria has been suggested (Lehenbauer-Baum and Fohringer, 2015, Lehenbauer-Baum et al., 2015, Snodgrass et al., 2019). Previous research describes how the criteria tolerance, mood modification and cognitive salience correlates stronger to engagement and the criteria, here termed “core criteria”; withdrawal, relapse, conflict and problems correlates stronger to addiction (Brunborg et al., 2013, Brunborg et al., 2015, Charlton and Danforth, 2007). This study presents a prevalence measure in the Swedish population of highly engaged gamers, problem gamers and addicted gamers using the core approach. The study also shows how these groups of gamers differ. Male gender, young age, social satisfaction, treatment need and hours chatting on internet/social media was shown to increase the probability of problematic or addictive gaming, whereas no difference was seen regarding young age, social satisfaction or treatment need when compared directly with the highly engaged gamers.

The present study showed that the prevalence of addicted gamers was 1.2 percent. The most precise prevalence worldwide, reported as 3.05 percent by Stevens et al., was indeed higher though not entirely comparable - their meta-analysis only included studies with youths and young adults. Their numbers are, however comparable with the prevalence presented within the younger age categories in the present study (Ferguson et al., 2011). A Norwegian population-based investigation actually reported an identical prevalence rate of addicted gamers; 1.2 percent, whereas their prevalence of problem gamers was slightly higher (Brunborg, Hanss, Mentzoni, & Pallesen, 2015).

Male gender showed a disproportionate prevalence of problem gamers, in concordance with previous research (Gonzalez-Bueso et al., 2018, Mentzoni et al., 2011). The addicted gamers showed the same gender tendency though not significantly, while the opposite applied for the highly engaged gamers but again not significantly. The group called RSP showed no gender difference which could suggest that men and women in the Swedish population overall engage in excessive gaming to an analogous extent, but that gaming implies a greater pathological potential for men. In 2015 Brunborg et al. presented a comparable Norwegian study, also using the core approach (Brunborg et al., 2015). They consistently stated that engaged, problem and addicted gamers were more likely to be male (Wittek et al., 2015). Simultaneously, ESÁs bulletin of the computer and video game industry from 2007 reports on 38 percent female gamers while the equivalent from 2020 reports on 41 percent female gamers (Entertainment Software Association, 2007, Entertainment Software Association, 2018). Perhaps, as the demographics in gaming patterns change, so will the gender differences regarding problematic gaming. Additional research is needed to widen the understanding of gender-specific gaming behaviour.

The highly engaged gamers, as well as the problem gamers, were overrepresented amongst those assenting that they had considered seeking treatment for psychological health complaints. Pathological gaming has been associated to various psychosocial problems (Gonzalez-Bueso et al., 2018, Lam, 2014, Lemmens et al., 2011) and one could assume that the tendency noted within the groups of addicted gamers would have achieved a significant value if the population had been greater in number. On the other hand, the denial of such consideration should perhaps not automatically be interpreted as a guarantee of good psychological health. Even though it is possible to experience poor psychological well-being without considering seeking help, it is relevant that confirming treatment had been considered implied an increased probability of problematic or addictive gaming 1.9 times.

The feeling of having enough friends was shown to be negatively associated with problem gaming in accordance with previous research (Ferguson et al., 2011, Gonzalez-Bueso et al., 2018). Having enough friends decreased the probability of problematic or addictive gaming 0.65 times. Interestingly, the highly engaged gamers were also more likely to experience feelings of loneliness and a desire to have more friends. ESÁs bulletin of 2018 states that 55 percent of the most frequent gamers report that video games help connect them with their friends and that 46 percent communicate that gaming helps their family to spend time together (Entertainment Software Association, 2018). Perhaps the gaming could be interpreted as a way towards the goal of decreasing one’s loneliness.

As age increased, the frequency of both engaged and problem gamers was shown to decrease; this is not surprising as young age is usually reported as associated to game addiction (Mentzoni et al., 2011, Wittek et al., 2015). The probability of problematic or addictive gaming was consistently shown to decrease 0.65 times as the category increased by age.

The one factor showing the most powerful correlation with problematic or addictive gaming was, interestingly, spending more than four hours daily chatting on internet/social media, increasing the probability 5.3 times. This subgroup also showed a disproportionate prevalence of both engaged, problem, and addicted gamers. The original survey asked about hours chatting on internet/social media specified such as chat functions including online gaming, which could imply a self-fulfilling association. The relationship between social networking site addiction and internet gaming disorder has, however, been described in previous research, hypothetically due to an underlying commonality such as to connecting with friends, or as a consequence of traits predisposing for behavioural addiction (Andreassen et al., 2013, Pontes, 2017).

Previous research emphasizes a qualitative difference between engagement and addiction whereby addiction but not engagement is related to negative outcome(Snodgrass et al., 2019). Brunborg et al. described how the magnitude of problems gradually increased from engagement to problematic to addictive gaming(Brunborg et al., 2013). They reported that both problem gamers and addicted gamers (to different degrees), but not the engaged gamers, showed greater risk of irritability, nervousness, tiredness, exhaustion and feeling low, afraid or in bad mood. In direct comparison however, they found no difference in terms of sleep problems, fear or symptoms of depression between addicted, problem and engaged gamers. In the present study a comparison between the probability of problematic or addictive gaming with highly engaged gaming within the variables of interest, only male gender and hours spent chatting was shown to increase the probability of a greater intensity of gaming. Interestingly, there was no detectable difference in terms of considering seeking treatment or experiencing feelings of loneliness. Previous research reports addictive gamers as more likely to be lonely and less socially competent (Khazaal et al., 2016, Lemmens et al., 2011). The fact that the group that experienced feeling of loneliness in the present study showed a disproportionate prevalence of both engaged gamers and problem gamers suggests that also the engaged gamers also experience social difficulties. Regarding the need to seek treatment for poor psychological wellbeing, one could only speculate whether the group displaying more intense gaming behaviour would be less inclined to seek help, despite poorer psychological wellbeing, or whether the measures used failed to capture an actual discrepancy.

The material used for this study was collected during two different studies; the previous was intended to serve as a database for a pilot investigation and was collected via a survey spread as wide as possible, while the other selected respondents deliberately, aiming to distribute gender and age evenly. The latter material was based on respondents recruited via a web panel, which could imply a selection bias, so it is possible that some groups participated less frequently. The groups did differ in some regards; the first data set had a greater proportion aged 19–24 years of age as well as a greater number of problem gamers. However, both of the included data set used the measurement needed for the aimed prevalence estimate and were included considering our exploratory intention in a still rather unsatisfactory field of research. In terms of demographics such as gender and age, the final material was, however, representatively distributed and in order to avoid giving some groups a disproportionate impact no weighting was used. An additional limitation is the relatively excessive loss in terms of excluded individuals which could entail a selection bias. The excluded individuals were evenly distributed in terms of gender but the excluded population contained a greater proportion of the youngest individuals and less of the oldest individuals. As gaming is known to be more common among young individuals(Mentzoni et al., 2011, Wittek et al., 2015) this could result in an underrate of the overall prevalence estimates. The present study used the seven-item version of GAS, in order to shorten the survey in this online survey context as much as possible. Some gaming research applies instruments based on all of the nine proposed criteria from DSM-5^17.32^, which could be clinically favourably as they could be considered more direct applicable for APA’s internet gaming disorder criteria. Hence the choice to use the seven-item version of GAS could also be considered a limitation. However, GAS is an established, brief and well-studied instrument with psychometric properties that has been demonstrated as equal to instruments based on all of the nine DSM-5 criteria(Brunborg et al., 2013, Brunborg et al., 2015, Khazaal et al., 2016, King et al., 2020, Lemmens et al., 2009, Lin et al., 2019, Mentzoni et al., 2011, Wittek et al., 2015). Further, the cross-sectional design of this study obviously does not permit conclusions to be drawn regarding causation since such would require longitudinal investigation.

## 5 Conclusion

Given the occurrence of game addiction as a tentative clinical diagnosis, there are still unanswered questions to be addressed. There is still no established diagnostic cut off and the field of research consequently lacks consistency. Furthermore, additional longitudinal investigations are required in order to fully understand the causality of comorbidity. However, this study contributes with a prevalence measure of game addiction, problem gaming and engaged gaming using an approach aiming to distinguish the interfering features of gaming in order to avoid pathologizing a recreational activity. This study presents results that indicate that highly engaged gamers, problem gamers and addicted gamers experience loneliness and psychological distress to a greater extent than the remaining study participants.

## Ethical approval

All procedures performed in studies involving human participants were in accordance with the ethical standards of the institutional and/or national research committee and with the 1964 Helsinki declaration and its later amendments or comparable ethical standards.

**Consent for publication**

Consent for publication has been given by all participants in the study.

## Funding

The study was enabled through funding from Region Skåne; Craaford Foundation grant; Fanny Ekdahls stiftelse and Svenska spels forskningsråd

## CRediT authorship contribution statement

**Frida André:** Investigation, Visualization, Software, Methodology, Conceptualization, Writing - original draft, Data curation, Formal analysis, Writing - review & editing. **Niroshani Broman:** Investigation, Writing - review & editing, Resources. **Anders Håkansson:** Validation, Methodology, Conceptualization, Writing - review & editing, Project administration. **Emma Claesdotter-Knutsson:** Validation, Visualization, Software, Writing - original draft, Supervision, Writing - review & editing, Project administration.

## Declaration of Competing Interest

The authors declare that they have no known competing financial interests or personal relationships that could have appeared to influence the work reported in this paper.
